# Supplementary material for: Mutational Profile of Blood and Tumor Tissue and Biomarkers of Response to PD-1 Inhibitors in Patients with Cutaneous Squamous Cell Carcinoma
Source: Cancers (Basel). 2025 Mar 31;17(7):1172. doi: 10.3390/cancers17071172 (PMC11987913; doi:10.3390/cancers17071172)
Supplement: Supplementary file 1 [file cancers-17-01172-s001.zip › cancers-3485356-supplementary.pdf]

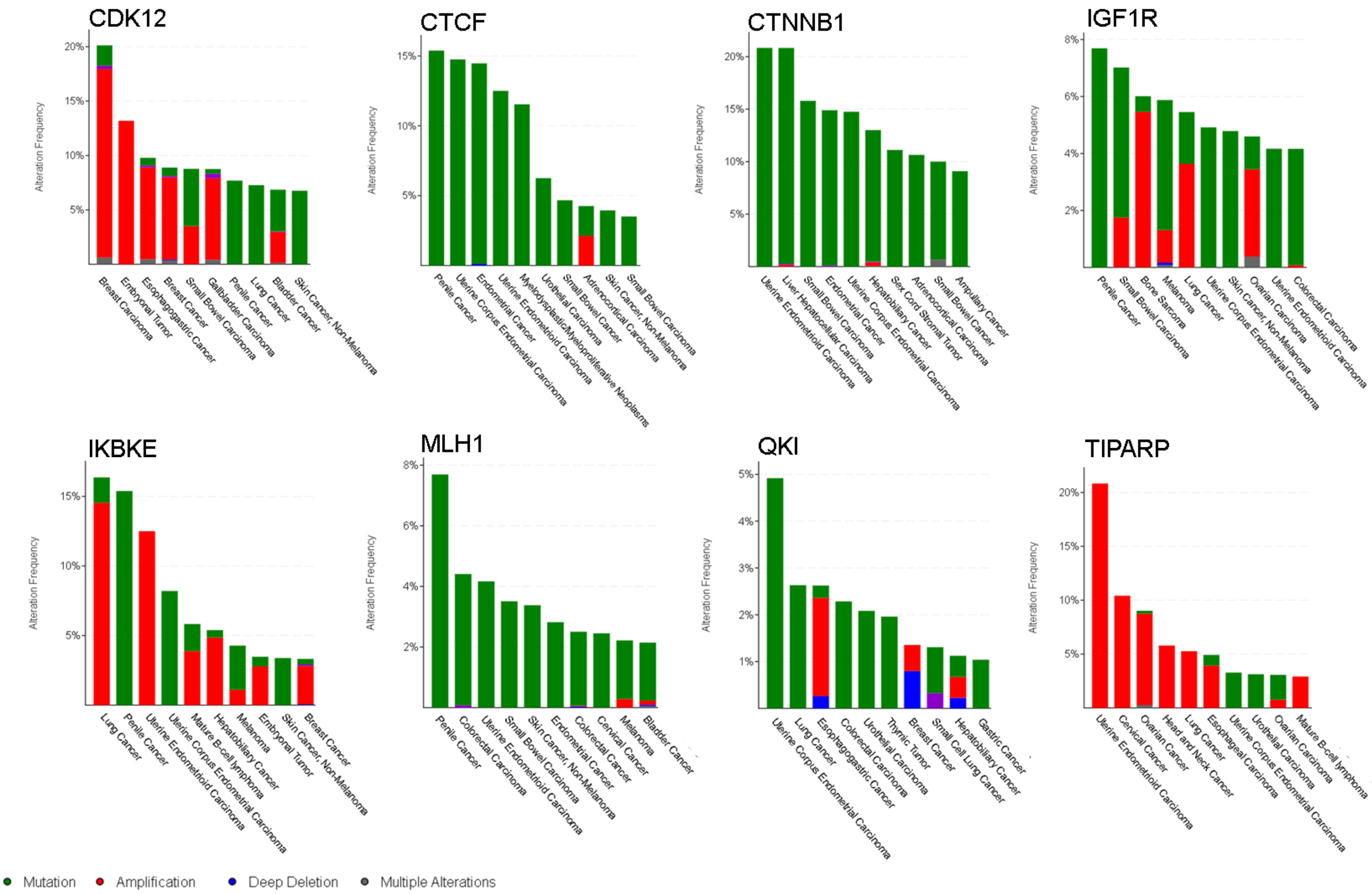

Figure S1: Incidence of gene alterations across various cancer types from PanCancer studies generated with cBioPortal software, https://www.cbioportal.org/ [32,47–59].

Table S1. Characterization of molecular alterations of the eight significant mutated genes in this cohort.

Tumor DNA

Gene of significance	Type of alteration	Alteration effect	Response
CDK12	AA alteration	Y246D	Non-responder
CDK12	AA alteration	P672S	Non-responder
CDK12	AA alteration	K371T	Responder
CDK12	AA alteration	P1273S	Non-responder
CDK12	AA alteration	T1337M	Non-responder
CDK12	AA alteration	K208E	Non-responder
CTCF	AA alteration	G47A	Non-responder
CTCF	AA alteration	P312H	Non-responder
CTNNB1	Nonsense mutation	Q322*	Non-responder
CTNNB1	AA alteration	R453W	Non-responder
IGF1R	AA alteration	M1098I	Non-responder
IGF1R	AA alteration	P835L	Non-responder
IGF1R	AA alteration	L863I	Non-responder
IGF1R	AA alteration	G540R	Responder
IGF1R	AA alteration	P323S	Responder
IGF1R	AA alteration	S729F	Responder
IKBKE	AA alteration	S315F	Non-responder
IKBKE	AA alteration	E448K	Non-responder
IKBKE	AA alteration	V311I	Non-responder
IKBKE	AA alteration	H583Y	Responder
IKBKE	AA alteration	S315F	Non-responder
MLH1	AA alteration	E736K	Non-responder
MLH1	Rearrangement		Non-responder
QKI	AA alteration	D16N	Non-responder
QKI	Frameshift mutation	K134fs	Non-responder
QKI	AA alteration	G278R	Non-responder
QKI	AA alteration	R53W	Responder
TIPARP	Nonsense mutation	Q499*	Non-responder
TIPARP	Amplification		Non-responder

Circulating tumor DNA

Gene of significance	Type of alteration	Alteration effect	Response
CDK12	AA alteration	S1072L	Responder
CDK12	AA alteration	K745K	Responder
CDK12	Frameshift mutation	P604fs	Non-responder
CDK12	AA alteration	P672S	Non-responder

AA, amino acid

(fs) = frameshift

(*) = stop codon

References

32. Samstein, R.M.; Lee, C.H.; Shoushtari, A.N.; Hellmann, M.D.; Shen, R.; Janjigian, Y.Y.; Barron, D.A.; Zehir, A.; Jordan, E.J.; Omuro, A.; et al. Tumor mutational load predicts survival after immunotherapy across multiple cancer types. *Nat. Genet.* **2019**, *51*, 202–206. https://doi.org/10.1038/s41588-018-0312-8.
47. Cerami, E.; Gao, J.; Dogrusoz, U.; Gross, B.E.; Sumer, S.O.; Aksoy, B.A.; Jacobsen, A.; Byrne, C.J.; Heuer, M.L.; Larsson, E.; et al. The cBio Cancer Genomics Portal: An Open Platform for Exploring Multidimensional Cancer Genomics Data. *Cancer Discov.* **2012**, *2*, 401–404.
48. Gao, J.; Aksoy, B.A.; Dogrusoz, U.; Dresdner, G.; Gross, B.; Sumer, S.O.; Sun, Y.; Jacobsen, A.; Sinha, R.; Larsson, E.; et al. Integrative analysis of complex cancer genomics and clinical profiles using the cBioPortal. *Sci. Signal.* **2013**, *6*, pl1.
49. de Bruijn, I.; Kundra, R.; Mastrogiacomo, B.; Tran, T.N.; Sikina, L.; Mazor, T.; Li, X.; Ochoa, A.; Zhao, G.; Lai, B.; et al. Analysis and Visualization of Longitudinal Genomic and Clinical Data from the AACR Project GENIE Biopharma Collaborative in cBioPortal. *Cancer Res.* **2023**, *83*, 3861–3867.
50. Jee, J.; Fong, C.; Pichotta, K.; Tran, T.N.; Luthra, A.; Waters, M.; Fu, C.; Altoe, M.; Liu, S.Y.; Maron, S.B.; et al. Automated real-world data integration improves cancer outcome prediction. *Nature* **2024**, *636*, 728–736. https://doi.org/10.1038/s41586-024-08167-5.
51. Zehir, A.; Benayed, R.; Shah, R.H.; Syed, A.; Middha, S.; Kim, H.R.; Srinivasan, P.; Gao, J.; Chakravarty, D.; Devlin, S.M.; et al. Mutational landscape of metastatic cancer revealed from prospective clinical sequencing of 10,000 patients. *Nat. Med.* **2017**, *23*, 703–713. https://doi.org/10.1038/nm.4333.
52. Robinson, D.R.; Wu, Y.M.; Lonigro, R.J.; Vats, P.; Cobain, E.; Everett, J.; Cao, X.; Rabban, E.; Kumar-Sinha, C.; Raymond, V.; et al. Integrative clinical genomics of metastatic cancer. *Nature* **2017**, *548*, 297–303. https://doi.org/10.1038/nature23306.
53. Miao, D.; Margolis, C.A.; Vokes, N.I.; Liu, D.; Taylor-Weiner, A.; Wankowicz, S.M.; Adeegbe, D.; Keliher, D.; Schilling, B.; Tracy, A.; et al. Genomic correlates of response to immune checkpoint blockade in microsatellite-stable solid tumors. *Nat. Genet.* **2018**, *50*, 1271–1281. https://doi.org/10.1038/s41588-018-0200-2.
54. Hyman, D.M.; Piha-Paul, S.A.; Won, H.; Rodon, J.; Saura, C.; Shapiro, G.I.; Juric, D.; Quinn, D.I.; Moreno, V.; Doger, B.; et al. HER kinase inhibition in patients with HER2- and HER3-mutant cancers. *Nature* **2018**, *554*, 189–194. https://doi.org/10.1038/nature25475.
55. Rosen, E.Y.; Goldman, D.A.; Hechtman, J.F.; Benayed, R.; Schram, A.M.; Cocco, E.; Shifman, S.; Gong, Y.; Kundra, R.; Solomon, J.P.; et al. TRK Fusions Are Enriched in Cancers with Uncommon Histologies and the Absence of Canonical Driver Mutations. *Clin. Cancer Res. Off. J. Am. Assoc. Cancer Res.* **2020**, *26*, 1624–1632. https://doi.org/10.1158/1078-0432.CCR-19-3165.
56. Bolton, K.L.; Ptashkin, R.N.; Gao, T.; Braunstein, L.; Devlin, S.M.; Kelly, D.; Patel, M.; Berthon, A.; Syed, A.; Yabe, M.; et al. Cancer therapy shapes the fitness landscape of clonal hematopoiesis. *Nat. Genet.* **2020**, *52*, 1219–1226. https://doi.org/10.1038/s41588-020-00710-0.
57. Wu, L.; Yao, H.; Chen, H.; Wang, A.; Guo, K.; Gou, W.; Yu, Y.; Li, X.; Yao, M.; Yuan, S.; et al. Landscape of somatic alterations in large-scale solid tumors from an Asian population. *Nat. Commun.* **2022**, *13*, 4264. https://doi.org/10.1038/s41467-022-31780-9.
58. The ICGC/TCGA Pan-Cancer Analysis of Whole Genomes Consortium. Pan-cancer analysis of whole genomes. *Nature* **2020**, *578*, 82–93. https://doi.org/10.1038/s41586-020-1969-6.
59. Nguyen, B.; Fong, C.; Luthra, A.; Smith, S.A.; DiNatale, R.G.; Nandakumar, S.; Walch, H.; Chatila, W.K.; Madupuri, R.; Kundra, R.; et al. Genomic characterization of metastatic patterns from prospective clinical sequencing of 25,000 patients. *Cell* **2022**, *185*, 563–575.e11. https://doi.org/10.1016/j.cell.2022.01.003.
